# Influence of anterior mitral valve length and septal wall thickness on the prevalence of left ventricular outflow tract obstruction in hypertrophic cardiomyopathy

**DOI:** 10.1186/1532-429X-14-S1-P169

**Published:** 2012-02-01

**Authors:** Kareem Morant, John Stirrat, David Scholl, Maria Drangova, James A White

**Affiliations:** 1 Deparment of Medicine, University of Western Ontario, London, ON, Canada; 2 Division of Cardiology, Department of Medicine, London Health Sciences Centre, London, ON, Canada; 3 Imaging Research Labratory-Robarts Research Institute, University of Western Ontario, London, ON, Canada

## Summary

In this study, we aimed to identify the respective and combined influences of AMVL length and septal thickening on the development of LVOT obstruction in a population of patients with HCM.

## Background

Interest has emerged in the evaluation of mitral valve morphology in patients with Hypertrophic Cardiomyopathy (HCM) and its contribution to left ventricular outflow tract obstruction (LVOTO). Specifically, elongation of the anterior mitral valve leaflet (AMVL) appears to be part of phenotypic expression in HCM and has been associated with severity of LVOT obstruction. However, the influence of septal thickening and its physiologic interaction with AMVL elongation is not well understood.

## Methods

Consecutive HCM patients referred for Cardiac MRI evaluation between March 2008 and May 2011 were identified from a prospective clinical registry. All patients underwent a standardized imaging protocol inclusive of cine imaging and phase-contrast flow imaging. Cine imaging was performed in sequential short axis planes and in the 2, 3 and 4-chamber views. Phase contrast flow of the LVOT was performed to identify the peak systolic velocity. All images were analyzed using commercially available software (CMR42, Circle Cardiovascular Inc., Calgary). Cine images were blindly analyzed for AMVL length, septal wall thickness (SWT), LVOT diameter, LV chamber volumes and stroke volume. A novel calculated measure was also evaluated, the “Septal Anterior Mitral Valve Length Product” (SALP), determined by the multiplying of SWT and AMVL length. All patients with an LVOT pressure gradient ≥30 mmHg were defined as having LVOTO.

## Results

**Figure 1:**
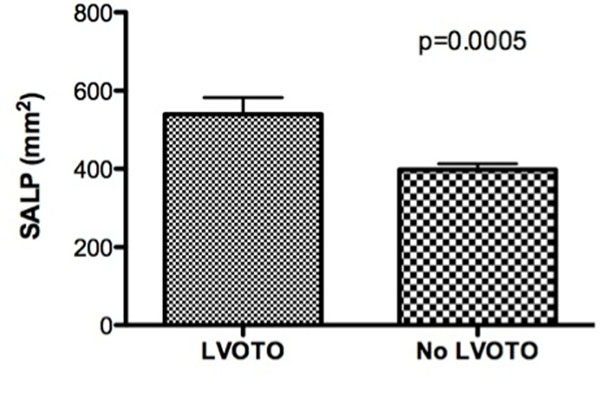


**Figure 2:**
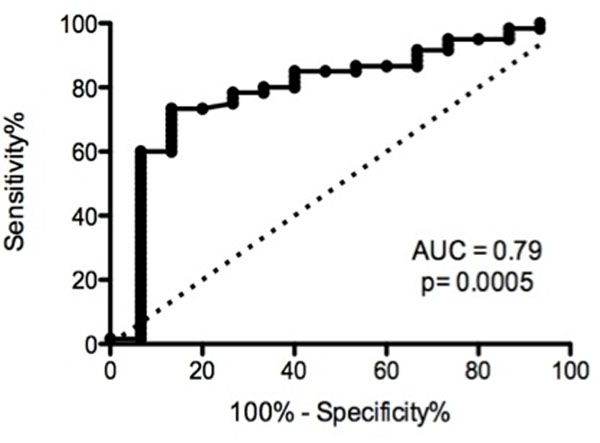


A total of 75 patients were studied with a mean age of 54 ± 14 years. The mean maximal wall thickness was 19.5 ± 5.5mm. Among all baseline clinical and MRI characteristics SWT and SALP were the only significant predictors of resting LVOTO, occurring in 15 (19.2%) patients (Figure 1). By Receiver Operator Characteristics (ROC) analysis SALP demonstrated superior predictive accuracy for the occurrence of LVOTO (AUC=0.79) (Figure 2) compared with SWT alone (AUC=0.71). The sensitivity and specificity of SALP for prediction of obstruction was 73% and 87%, respectively.

## Conclusions

This study identifies a combined influence of AMVL length and SWT on the development of LVOTO in HCM. The product of these two measures, a marker we term SALP, may be a useful tool to identify early clinical phenotypes of HCM and those at higher likelihood of dynamic LVOTO.

## Funding

J.A.W is a clinician scientist with the Heart and Stroke Foundation of Ontario, Canada. This research was supported by the Heart and Stroke Foundation grant # NA6488 (PI: J.A.W.) and by the Canada

Foundation of Innovation (CFI) Leaders Opportunity Fund.

